# Supplementary material for: MicroRNAs from Snellenius manilae bracovirus regulate innate and cellular immune responses of its host Spodoptera litura
Source: Commun Biol. 2021 Jan 8;4:52. doi: 10.1038/s42003-020-01563-3 (PMC7794284; doi:10.1038/s42003-020-01563-3)
Supplement: Supplementary file 2 — Supplementary Information [file 42003_2020_1563_MOESM2_ESM.pdf]

**MicroRNAs from *Snellenius manilae* bracovirus regulate innate and cellular immune responses of its host *Spodoptera litura***

Cheng-Kang Tang, Chih-Hsuan Tsai, Carol-P Wu, Yu-Hsien Lin, Sung-Chan Wei, Yun-Heng Lu, Cheng-Hsun Li, and Yueh-Lung Wu*
Department of Entomology, National Taiwan University, Taipei 106, Taiwan

**Supplementary Figures and Tables and Materials and Methods**

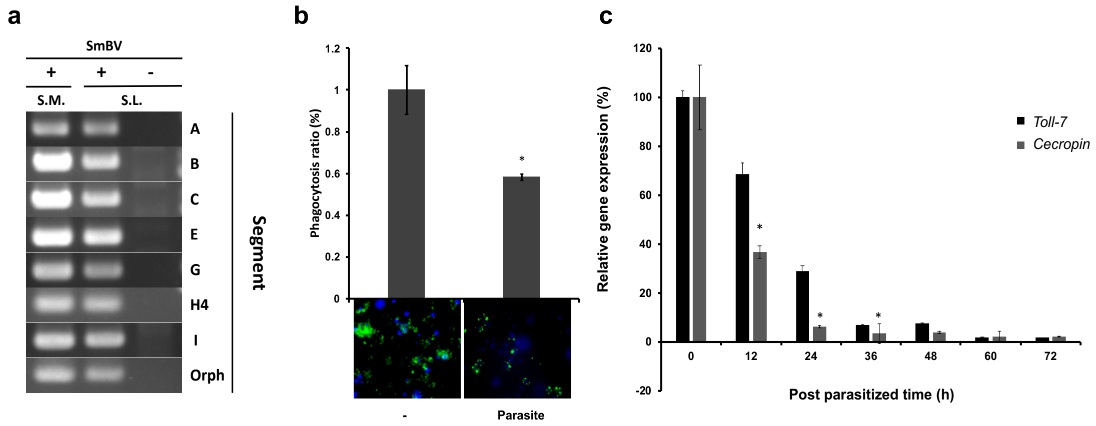


**Supplementary Figure 1. Identification of PDV genome segments and host immune responses in *S. litura* second-instar larvae parasitized by *S. manilae*. a** Identification of SmBV genome segments in *S. manilae* ovaries and parasitized *S. litura*. We extracted the total RNA from the ovaries of female *S. manilae* (S.M., the first lane), *S. litura* second-instar larvae parasitized by *S. manilae* (S.L., the second lane), and wild type *S. litura* second-instar larvae without SmBV infection (S.L., the third lane). The SmBV genome was determined as segments including A, B, C, E, G, H4, I, and Orph. **b** Phagocytosis assay of second-instar *S. litura* larvae with or without parasitism by *S. manilae* wasps for 36 h. The phagocytosis ratio (%) = FITC / DAPI, in which the FITC is green fluorescence detected from FITC-labeled *E. coli* and DAPI is blue fluorescence detected from cells. **c** Determination of the expression of *Toll-7* and *Cecropin* genes in *S. litura* 2$^{nd}$ instar larvae parasitized by *S. manilae*. Relative expression levels of *toll-7* and *cecropin* in Toll pathway were determined by qPCR from 0 to 72 h after *S. manilae* parasitism. The expressions were normalized with that of *18S* rRNA. Black bars represent the *Toll-7* gene and grey bars represent the *Cecropin* gene. All experiments were performed with three biological replicates. Data are expressed as the mean and standard deviation (SD). *p*-values were calculated using Student's t-test (*, $p < 0.05$).

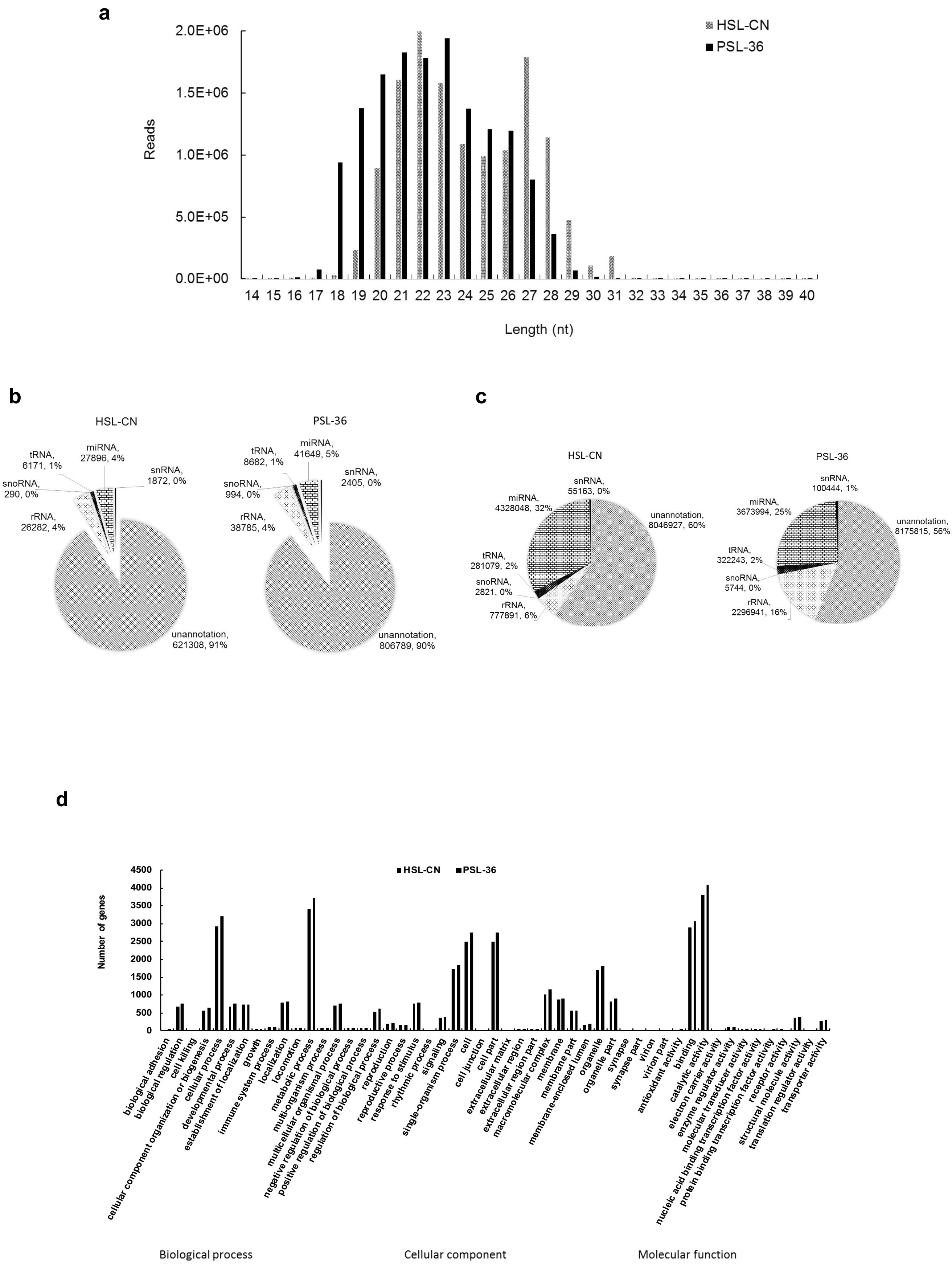
a
HSL-CN
PSL-36
Reads
Length (nt)
2.0E+06
1.5E+06
1.0E+06
5.0E+05
0.0E+00
14 15 16 17 18 19 20 21 22 23 24 25 26 27 28 29 30 31 32 33 34 35 36 37 38 39 40
b
HSL-CN
tRNA, 6171, 1%
miRNA, 27896, 4%
snRNA, 1872, 0%
snoRNA, 290, 0%
rRNA, 26282, 4%
unannotation, 621308, 91%
PSL-36
tRNA, 8682, 1%
miRNA, 41649, 5%
snRNA, 2405, 0%
snoRNA, 994, 0%
rRNA, 38785, 4%
unannotation, 806789, 90%
c
HSL-CN
snRNA, 55163, 0%
miRNA, 4328048, 32%
unannotation, 8046927, 60%
tRNA, 281079, 2%
snoRNA, 2821, 0%
rRNA, 777891, 6%
PSL-36
snRNA, 100444, 1%
miRNA, 3673994, 25%
unannotation, 8175815, 56%
tRNA, 322243, 2%
snoRNA, 5744, 0%
rRNA, 2296941, 16%
d
HSL-CN
PSL-36
Number of genes
4500
4000
3500
3000
2500
2000
1500
1000
500
0
biological adhesion
biological regulation
cell killing
cellular component organization or biogenesis
cellular process
developmental process
establishment of localization
growth
immune system process
localization
locomotion
metabolic process
multi-organism process
multicellular organismal process
negative regulation of biological process
positive regulation of biological process
regulation of biological process
reproduction
reproductive process
response to stimulus
rhythmic process
signaling
single-organism process
cell
cell junction
cell part
extracellular matrix
extracellular region
extracellular region part
macromolecular complex
membrane
membrane part
membrane-enclosed lumen
organelle
organelle part
synapse
synapse part
virion
virion part
antioxidant activity
binding
catalytic activity
electron carrier activity
enzyme regulator activity
molecular transducer activity
nucleic acid binding transcription factor activity
protein binding transcription factor activity
receptor activity
structural molecule activity
translation regulator activity
transporter activity
Biological process
Cellular component
Molecular function

**Supplementary Figure 2. Characterization of small RNA contents of non-infected *S. litura* second-instar larvae and *S. litura* larvae parasitized by *S. manilae* for 36 h. a** Length distribution of small RNA raw reads from *S. litura* non-infected second-instar larvae (HSL-CN, grey bars) and *S. litura* second-instar larvae parasitized by *S. manilae* for 36 h (PSL-36, black bars). **b** Small RNA raw reads detected by small RNA Hiseq next generation sequencing from non-infected *S. litura* second-instar larvae (HSL-CN) and those parasitized by *S. manilae* for 36 h (PSL-36). The percentage represents the amount of a certain small RNA within the whole small RNA categories. **c** Small RNA clean reads with the removal of adaptor sequences and contaminated reads. The percentage represents the amount of a certain small RNA within the whole small RNA categories. **d** Gene ontology (GO) analysis of predicted target genes of newly identified small RNAs. The target genes were predicted for novel small RNAs identified from HSL-CN (grey bars) and PSL-36 (black bars) and characterized by their involved biological processes, cellular component, and molecular functions.

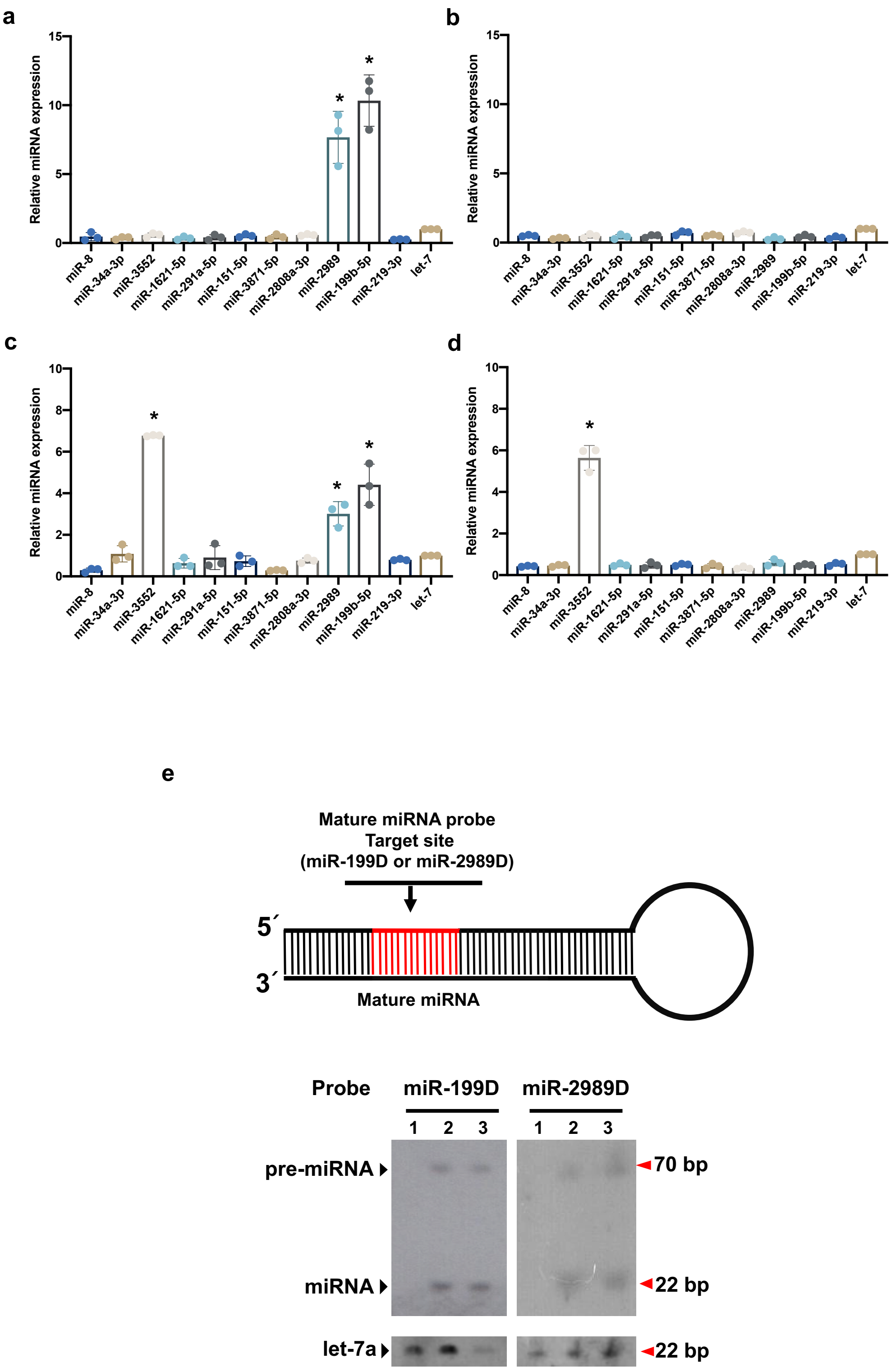
a
b
c
d
Relative miRNA expression
miR-8
miR-34a-3p
miR-3552
miR-1621-5p
miR-291a-5p
miR-151-5p
miR-3871-5p
miR-2808a-3p
miR-2989
miR-199b-5p
miR-219-3p
let-7
e
Mature miRNA probe
Target site
(miR-199D or miR-2989D)
5´
3´
Mature miRNA
Probe
miR-199D
miR-2989D
1
2
3
pre-miRNA
miRNA
let-7a
70 bp
22 bp
22 bp

**Supplementary Figure 3. Expression levels of different miRNAs in *S. litura* infected with SmBV, female wasp, or *S. litura* with or without wasp parasitism.** The expression levels of 11 predicted miRNAs were quantified using stem-loop qPCR in **a** *S. litura* with SmBV infection, **b** female wasp alone**, c** *S. litura* with or **d** without wasp parasitism were analyzed. The expression of *let-7* miRNA serves as a positive control. **e** Small RNAs harvested from *S. litura* with or without wasp parasitism or *S. litura* infected with SmBV were analyzed by Northern blotting using probes against mature and precursors miRNA (top panels). *let-7a* miRNA signal served as a positive control (bottom panels). 1: *S. litura* only; 2: *S. litura* with wasp parasitism; 3: *S. litura* infected with SmBV. At least three independent measurements were conducted for each group. A *Ct* value of 35 was set to be the cutoff for detection. Statistical analysis was performed using Student's t-test with $p$-values less than 0.05 (*, $p$ <0.05) considered statistically significant.

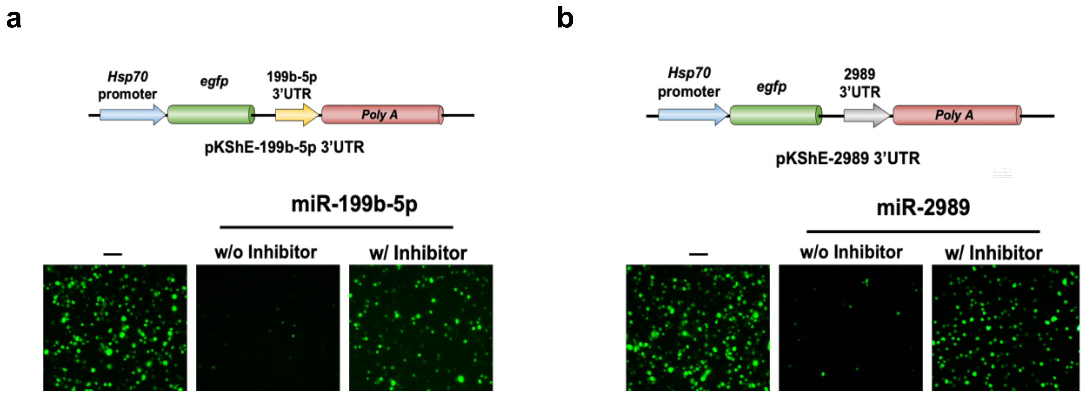


**Supplementary Figure 4. The specificity between miRNA mimic and its miRNA inhibitor.** Reporter plasmids pKShE-199b-5p 3'UTR **a** and pKShE-2989 3'UTR **b** in which the 3'-UTR of Domeless or Toll-7 were fused to the 3'-end of an EGFP coding region, respectively. Reporter plasmids were transfected into SL1A cells with miRNA mimic only or miRNA mimic plus its inhibitor. EGFP expression was detected at 48 hours after transfection by fluorescent microscopy.

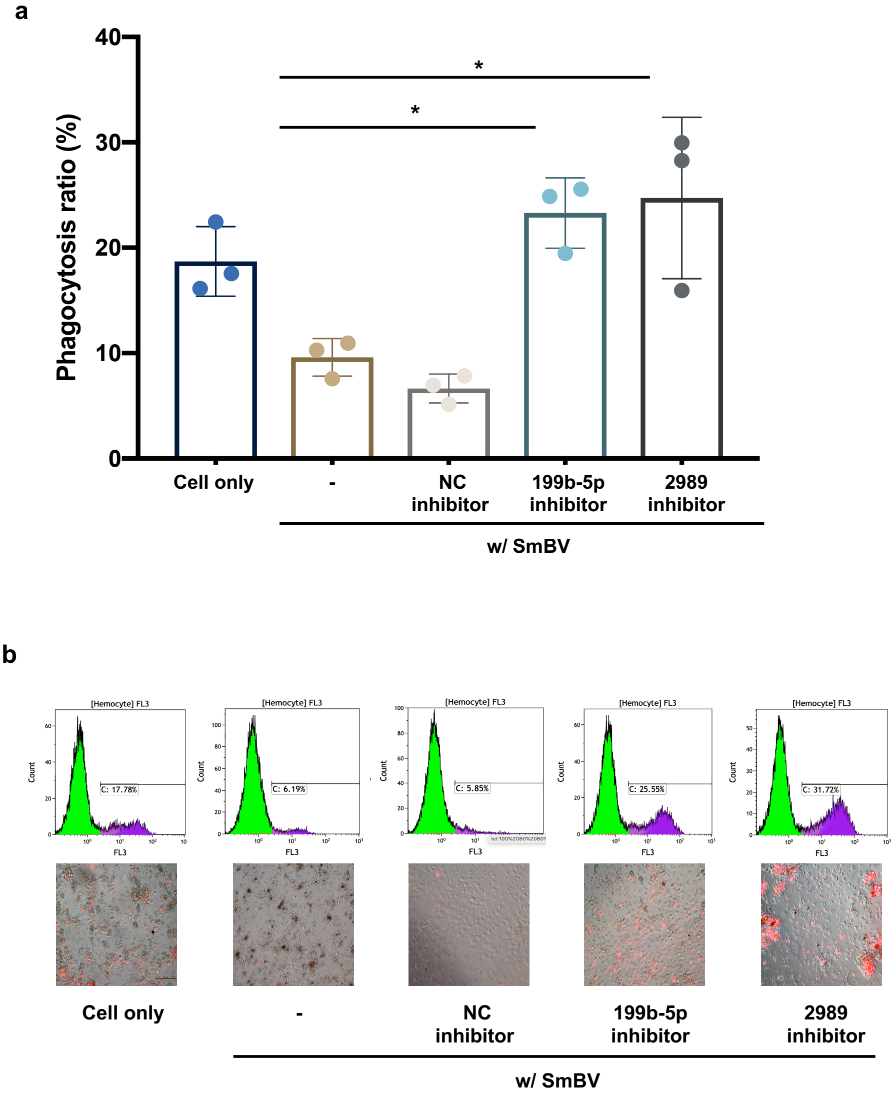


**Supplementary Figure 5. Inhibition of SmBV miRNA expression increases phagocytosis responses in *S. litura.*** Third-instar *S. litura* larvae were injected with SmBV, miR-199b-5p- or miR-2989 inhibitor and hemocytes were extracted 36 h after injection. The hemocytes were mixed with pHrodo™ Red dye conjugated *E. coli* and the phagocytosis activity of hemocytes was determined by flow cytometry. **a** The percentage of phagocytic cells (%) was derived from the proportion of pHrodo™ Red dye conjugated hemocytes. **b** Upper: representative histograms showing the percentage of red-fluorescence-positive cells from the phagocytosis activity of hemocytes. Lower: labeled *E. coli* and hemocytes were observed using fluorescence microscopy. All experiments were performed with four biological replicates. Data are expressed as the mean ± standard deviation (SD). *p*-values were calculated using Student's t-test (*, $p < 0.05$).

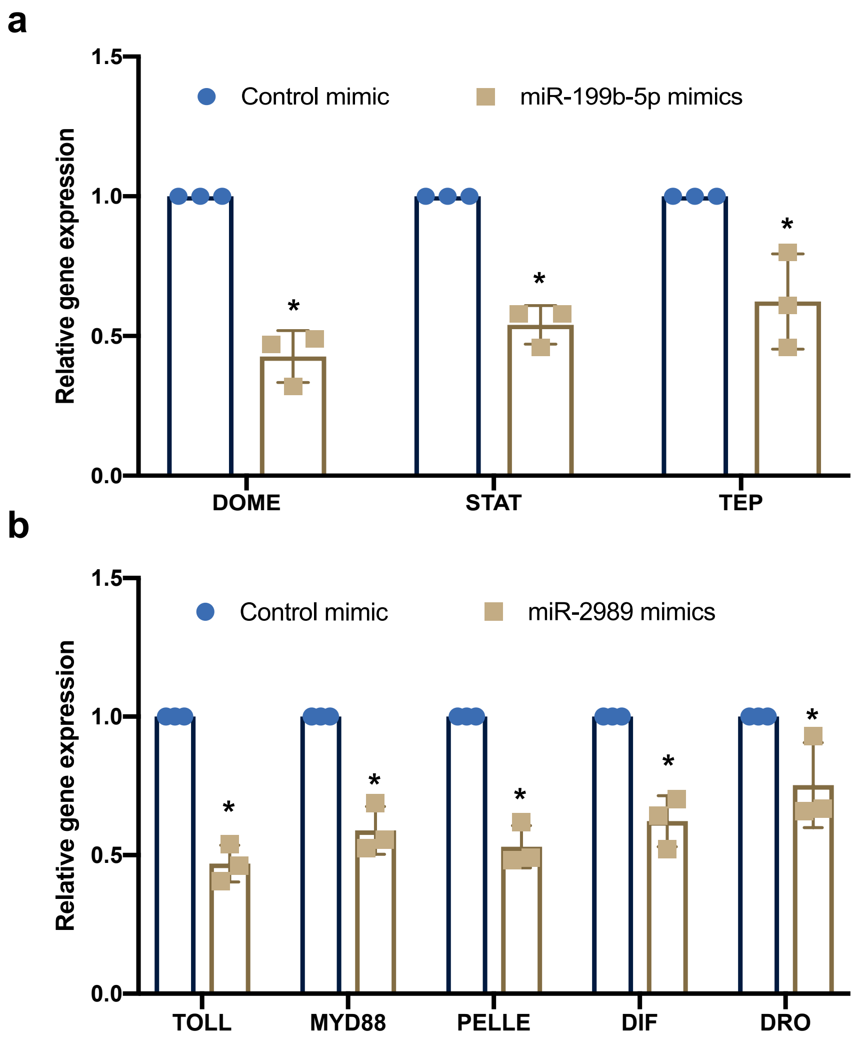


**Supplementary Figure 6. miR-199b-5p and miR-2989 mimics suppress the humoral immunity pathway in *S. litura*.** Genes in JAK/STAT **a** and Toll **b** pathways decreased in expression in third-instar *S. litura* larvae after the injection of miR-199b-5p and miR-2989 mimics, respectively. The expression levels of genes in the signal cascade of the JAK/STAT pathway [*domeless* (DOME), *STAT* (STAT), and *TEP* (TEP)] and the Toll pathway [*toll* (TOLL), *myd88* (MYD88), *pelle* (PELLE), *dif* (DIF), and *drosomycin* (DRO)] were determined by qPCR in *S. litura* larvae injected with miRNA mimics of miR-199b-5p and miR-2989, respectively. An *18s rRNA* signal was used as an internal control to normalize all readings. All experiments were performed with three biological replicates. Data are expressed as the mean and standard deviation (SD). *p*-values were calculated using Student's t-test (*, $p$ <0.05).

**a**

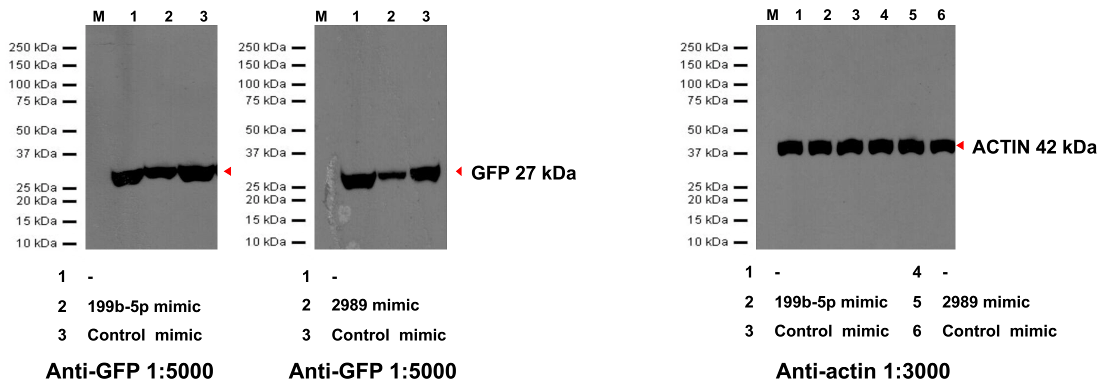


**Supplementary Fig. 7. The full, uncropped blot/gel images. a** Figure 2d.

**Supplementary Table 1. Sequences of miRNA candidates for subsequent experiments**

| miR_name | Reference miRNA ID | Accession number | miR-sequence |
|---|---|---|---|
| SmBV-miR-2989 | tgu-miR-2989 | MIMAT0014525 | 5’-GCACGUGAUGAGAACUCUGU-3’ |
| SmBV-miR-199b-5p | eca-miR-199b-5p | MIMAT0013780 | 5’-CCCAGUGUUUAGACUAUCUGUUC-3’ |
| SmBV-miR-219-3p | sja-miR-219-3p | MIMAT0016268 | 5’-UGAUUGUCCAUUCGCAUUUCU-3’ |
| SmBV-miR-8 | nvi-miR-8 | MIMAT0015698 | 5’-UAAUACUGUCAGGUAAAGAUGUC-3’ |
| SmBV-miR-3871-5p | tca-miR-3871-5p | MIMAT0018834 | 5’-GAUUUCUGCCUUGUGCCGC-3’ |
| SmBV-miR-3552 | mmu-miR-3552 | MIMAT0035715 | 5’-AGGCUGCAGGCCCACUUCCCU-3’ |
| SmBV-miR-151-5p | cgr-miR-151-5p | MIMAT0023782 | 5’-UCGAGGAGCUCACAGUCUAGU-3’ |
| SmBV-miR-34a-3p | str-miR-34a-3p | MIMAT0033485 | 5’-ACAGCUCACUCAACUGCCAAG-3’ |
| SmBV-miR-2808a-3p | bmo-miR-2808a-3p | MIMAT0013712 | 5’-CGGUGGUAGAUUCUGCGAAGUACG-3’ |
| SmBV-miR-1621-5p | gga-miR-1621-5p | MIMAT0007488 | 5’-ACCGGCUGCCUCGGUGGCAC-3’ |
| SmBV-miR-291a-5p | mmu-miR-291a-5p | MIMAT0000367 | 5’-CAUCAAAGUGGAGGCCCUCUCU-3’ |

**Supplementary Table 2. The list of miRNA Stem-loop primers**

| miR_name | Stem-loop primer |
|---|---|
| SmBV-miR-2989 | 5’-GTCGTATCCAGTGCAGGGTCCGAGG TATTCGCACTGGATACGACACAGAG-3’ |
| SmBV-miR-199b-5p | 5’-GTCGTATCCAGTGCAGGGTCCGAGG TATTCGCACTGGATACGACGAACAG-3’ |
| SmBV-miR-219-3p | 5’-GTCGTATCCAGTGCAGGGTCCGAGG TATTCGCACTGGATACGACTATGAT-3’ |
| SmBV-miR-8 | 5’-GTCGTATCCAGTGCAGGGTCCGAGG TATTCGCACTGGATACGACGACATC-3’ |
| SmBV-miR-3871-5p | 5’-GTCGTATCCAGTGCAGGGTCCGAGG TATTCGCACTGGATACGACGCGGCA-3’ |
| SmBV-miR-3552 | 5’-GTCGTATCCAGTGCAGGGTCCGAGG TATTCGCACTGGATACGACAGGGAA-3’ |
| SmBV-miR-151-5p | 5’-GTCGTATCCAGTGCAGGGTCCGAGG TATTCGCACTGGATACGACACTAGA-3’ |
| SmBV-miR-34a-3p | 5’-GTCGTATCCAGTGCAGGGTCCGAGG TATTCGCACTGGATACGACCTTGGC-3’ |
| SmBV-miR-2808a-3p | 5’-GTCGTATCCAGTGCAGGGTCCGAGG TATTCGCACTGGATACGACCGTACT-3’ |
| SmBV-miR-1621-5p | 5’-GTCGTATCCAGTGCAGGGTCCGAGG TATTCGCACTGGATACG ACGTGCCA-3’ |
| SmBV-miR-291a-5p | 5’-GTCGTATCCAGTGCAGGGTCCGAGG TATTCGCACTGGATACGACAGAGAG-3’ |

**Supplementary Table 3. The list of qPCR primers**

| Target gene name | qPCR forward primer |
|---|---|
| SmBV-miR-2989 | F: 5'-AATTGGCGCACGTGATGAG-3'<br>R: 5'-CCAGTGCAGGGTCCGAGGTA-3' |
| SmBV-miR-199b-5p | F: 5'-AATTTGGCGCCCAGTGTTTAG-3'<br>R: 5'-CCAGTGCAGGGTCCGAGGTA-3' |
| SmBV-miR-219-3p | F: 5'-AATGGCGGCAGAGGTGTTAAT-3'<br>R: 5'-CCAGTGCAGGGTCCGAGGTA-3' |
| SmBV-miR-8 | F: 5'-AATCGCGCGGCTAATACTGTCAG-3'<br>R: 5'-CCAGTGCAGGGTCCGAGGTA-3' |
| SmBV-miR-3871-5p | F: 5'-AATCGCGGCGATTTCTGCCTT-3'<br>R: 5'-CCAGTGCAGGGTCCGAGGTA-3' |
| SmBV-miR-3552 | F: 5'-AATGCAGGCTGCAGGCC-3'<br>R: 5'-CCAGTGCAGGGTCCGAGGTA-3' |
| SmBV-miR-151-5p | F: 5'-AATGCGCTCGAGGAGCTCA-3'<br>R: 5'-CCAGTGCAGGGTCCGAGGTA-3' |
| SmBV-miR-34a-3p | F: 5'-AATGCGGCACAGCTCACTCA-3'<br>R: 5'-CCAGTGCAGGGTCCGAGGTA-3' |
| SmBV-miR-2808a-3p | F: 5'-AATGCGGCCGGTGGTAGATT-3'<br>R: 5'-CCAGTGCAGGGTCCGAGGTA-3' |
| SmBV-miR-1621-5p | F: 5'-AATGCACCGGCTGCCTC-3'<br>R: 5'-CCAGTGCAGGGTCCGAGGTA-3' |
| SmBV-miR-291a-5p | F: 5'-AATGGCGGCCATCAAAGTGGA-3'<br>R: 5'-CCAGTGCAGGGTCCGAGGTA-3' |
| Let-7-5p | F: 5'-AAGCGCGCTGAGGTAGTAGG-3'<br>R: 5'-CCAGTGCAGGGTCCGAGGTA-3' |
| Toll-7 | F: 5'-AAGCGAAAGTTGGGGAATTT-3'<br>R: 5'-AGCTCCTTGAAAGTGCGGTA-3' |
| Myd88 | F: 5'-CAAAGAGAGGTAGTTAGGCCAAAAA-3'<br>R: 5'-GGTTTCTTTGGTTCCTTATCTTGAGA-3' |
| Pelle | F: 5'-TAACCGCCGAGATCCCAGAA-3'<br>R: 5'-GTTGTGCTGGTGCGATTGTT-3' |
| Dif | F: 5'-GTCGACCGCTGGATAGGAAG-3'<br>R: 5'-ACAAAGCAAAGTGTTCCGCC-3' |
| Drosomycin | F: 5'-CATTTACCAAGCTCCGTGAGAAC-3'<br>R: 5'-GGAGAGCTAAACGCGCTTTTCAG-3' |
| Domeless | F: 5'-ACAACAGGCGTCTTCGGATT-3'<br>R: 5'-ACCCTTCAGTTTTGCCATGGT-3' |
| Stat | F: 5'-TTCTGGGAGTGGTTCTACATGGT-3'<br>R: 5'-CTGGATGAAGCCCATGATCA-3' |
| TEP | F: 5'-AACTCCGCAAACACCAAGTTGG-3'<br>R: 5'-CTTCAACGCTTGGTGTAACACCA-3' |
| 18S rRNA | F: 5'-GTAACCCGTTGAACCCCATT-3'<br>R: 5'-CCATCCAATCGGTAGTAGCG-3' |

## Supplementary Methods

Northern blotting

Total small RNAs were extracted from *S. litura* with SmBV infection, *S. litura* with or without wasp parasitism using a mirVana$^{TM}$ miRNA isolation kit (Ambion) according to the manufacturer's instructions. For Northern blot analysis of small RNAs, 1 μg of small RNA sample was fractionated by 15% denaturing polyacrylamide gel electrophoresis (PAGE) (acrylamide:bis ratio, 19:1) containing 8 M urea in 0.5 × TBE buffer. RNAs were transferred to a Hybond-N+ nylon transfer membrane (GE Healthcare) by electroblotting and UV cross-linked. RNA oligonucleotides carrying the reverse complementary sequence for candidate miRNAs, precursor region, or *let-7a* were end-labeled with DIG (MD Bio. TW) to high specific activity. Hybridizations and washes were carried out using DIG hybridization buffer according to the manufacturer's directions (Roche)[1]. Sequence details for probes used in Northern blot analysis are as follows:
miR-199D (5'-GAACAGATAGTCTAAACACTGGG-3'); miR-2989D (5'-ACAGAGTTCTCATCACGTGC-3').

**Phagocytosis assays by flow cytometry**

Third-instar larvae were microinjected with SmBV (1 x $10^6$ virus copy number). Hemocytes were extracted 36 h post-injection and seeded onto a 96-well plate (4 × $10^4$ cells/well). Heat-killed *E. coli* labeled with the pH-dependent Red dye pHrodo (Thermo Fisher Scientific) (1 × $10^4$ cells/well) were then added[2]. After incubation, cells were collected and washed in cold PBS for flow-cytometric analysis. Uptake of pHrodo-labeled phagocyte was measured on a Galio flow cytometer (Beckman Coulter), and analyzed by using Kaluza Analysis 2.1. Ten thousand (1 x $10^4$) cells were analyzed per sample.

**Supplementary References**

1 Wu, P. C. *et al.* MicroRNAs derived from the insect virus HzNV-1 promote lytic infection by suppressing histone methylation. *Sci Rep* **8**, 17817, doi:10.1038/s41598-018-35782-w (2018).

2 Cho, Y. & Cho, S. Hemocyte-hemocyte adhesion by granulocytes is associated with cellular immunity in the cricket, Gryllus bimaculatus. *Sci Rep* **9**, 18066, doi:10.1038/s41598-019-54484-5 (2019).
