# Supplementary material for: MicroRNAs from Snellenius manilae bracovirus regulate innate and cellular immune responses of its host Spodoptera litura
Source: Commun Biol. 2021 Jan 8;4:52. doi: 10.1038/s42003-020-01563-3 (PMC7794284; doi:10.1038/s42003-020-01563-3)
Supplement: Supplementary file 3 — Description of Additional Supplementary Files [file 42003_2020_1563_MOESM3_ESM.docx]

**Description of Additional Supplementary Files：**

**File Name：**

**Supplementary Data1**

**Description:** Source data for the NGS (The miRNAs identified in SmBV and *S. litura*)

**Supplementary Data 2**

**Description:** Source data for the plots.
