# Supplementary material for: MicroRNAs from Snellenius manilae bracovirus regulate innate and cellular immune responses of its host Spodoptera litura
Source: Commun Biol. 2021 Jan 8;4:52. doi: 10.1038/s42003-020-01563-3 (PMC7794284; doi:10.1038/s42003-020-01563-3)
Supplement: Supplementary file 6 — Reporting Summary [file 42003_2020_1563_MOESM6_ESM.pdf]

nature research

Corresponding author(s): COMMSBIO-19-1955B Yueh-Lung Wu

Last updated by author(s): Sep 14, 2020

Reporting Summary

Nature Research wishes to improve the reproducibility of the work that we publish. This form provides structure for consistency and transparency in reporting. For further information on Nature Research policies, see Authors & Referees and the Editorial Policy Checklist.

Statistics

For all statistical analyses, confirm that the following items are present in the figure legend, table legend, main text, or Methods section.

n/a	Confirmed	
☐	☒	The exact sample size (*n*) for each experimental group/condition, given as a discrete number and unit of measurement
☐	☒	A statement on whether measurements were taken from distinct samples or whether the same sample was measured repeatedly
☐	☒	The statistical test(s) used AND whether they are one- or two-sided<br>*Only common tests should be described solely by name; describe more complex techniques in the Methods section.*
☒	☐	A description of all covariates tested
☐	☒	A description of any assumptions or corrections, such as tests of normality and adjustment for multiple comparisons
☐	☒	A full description of the statistical parameters including central tendency (e.g. means) or other basic estimates (e.g. regression coefficient) AND variation (e.g. standard deviation) or associated estimates of uncertainty (e.g. confidence intervals)
☒	☐	For null hypothesis testing, the test statistic (e.g. *F*, *t*, *r*) with confidence intervals, effect sizes, degrees of freedom and *P* value noted<br>*Give P values as exact values whenever suitable.*
☒	☐	For Bayesian analysis, information on the choice of priors and Markov chain Monte Carlo settings
☒	☐	For hierarchical and complex designs, identification of the appropriate level for tests and full reporting of outcomes
☒	☐	Estimates of effect sizes (e.g. Cohen's *d*, Pearson's *r*), indicating how they were calculated

Our web collection on statistics for biologists contains articles on many of the points above.

Software and code

Policy information about availability of computer code

Data collection	No software was used.
Data analysis	No software was used.

For manuscripts utilizing custom algorithms or software that are central to the research but not yet described in published literature, software must be made available to editors/reviewers. We strongly encourage code deposition in a community repository (e.g. GitHub). See the Nature Research guidelines for submitting code & software for further information.

Data

Policy information about availability of data

All manuscripts must include a data availability statement. This statement should provide the following information, where applicable:

- Accession codes, unique identifiers, or web links for publicly available datasets
- A list of figures that have associated raw data
- A description of any restrictions on data availability

All the experimental data and results could be examined and they are fully available to study.

Field-specific reporting

Please select the one below that is the best fit for your research. If you are not sure, read the appropriate sections before making your selection.

☒ Life sciences ☐ Behavioural & social sciences ☐ Ecological, evolutionary & environmental sciences

For a reference copy of the document with all sections, see nature.com/documents/nr-reporting-summary-flat.pdf

Life sciences study design

All studies must disclose on these points even when the disclosure is negative.

Sample size	In our experiments, we didn't use any statistical methods to predetermine sample size. But all the sample size that we determined were following the reference or get from related experimental protocols. We believe that should be enough to ensure statistical power of detection.
Data exclusions	No data were excluded from analysis. We had preliminary tests for each different experiments for getting the most appropriate experimental conditions. Therefore, after we got the conditions, we would not exclude the data from analysis.
Replication	The attempts at replication were successful. All experimental data that we shown were using the biological replicates. We didn't use the technical replicates in our results.
Randomization	In our study, we had a large amount of S. litura larvae. When we performed the experiments, we were all randomly pick the larvae from a pool and to become our target population. Within this target population, we would divided them into an equal amount of larvae for each treatment group. As this way, we could get a randomized experimental group.
Blinding	In our experiments, the blinding was not possible in our study.

Reporting for specific materials, systems and methods

We require information from authors about some types of materials, experimental systems and methods used in many studies. Here, indicate whether each material, system or method listed is relevant to your study. If you are not sure if a list item applies to your research, read the appropriate section before selecting a response.

Materials & experimental systems

n/a	Involved in the study	
☐	☒	Antibodies
☐	☒	Eukaryotic cell lines
☒	☐	Palaeontology
☐	☒	Animals and other organisms
☒	☐	Human research participants
☒	☐	Clinical data

Methods

n/a	Involved in the study	
☒	☐	ChIP-seq
☒	☐	Flow cytometry
☒	☐	MRI-based neuroimaging

Antibodies

Antibodies used	Anti-EGFP (Millipore): Anti-Green Fluorescent Protein Antibody, original GFP only, clone 264-449-2 (mouse monoclonal); Product No. MAB2510 (100 µL); LOT No. 3091231; EMD Millipore Corp., USA, +1-978-715-4321.<br>Anti-Actin (Millipore): Anti-Actin Antibody, clone C4 (mouse monoclonal); Product No. MAB1501 (100 µL); LOT No. 3209138; EMD Millipore Corp., USA, +1-978-715-4321.
Validation	Anti-EGFP (Millipore): Species: All; Application: Anti-Green Fluorescent Protein Antibody, original GFP only, clone 264-449-2 is an antibody against Green Fluorescent Protein for use in immunoprecipitation, western Blotting, and immunocytochemistry.; Specificity: 1.Directed against a bacterially-expressed fusion protein. 2.Also reacts with red-shifted GFP-variant. 3.Does not react with enhanced GFP.<br>Anti-Actin (Millipore): Species: All; Application: Reliably and specifically detect actin using this Anti-Actin Antibody, clone C4. This highly published monoclonal antibody is validated for use in ELISA, Immunocytochemistry, Immunofluorescence, Immunohistochemistry, Immunohistochemistry (Paraffin), and Western Blotting. This mAb is also available as a fluorescent conjugate.; Specificity: MAB1501 is a pan-actin antibody that binds to an epitope in a highly conserved region of actin; therefore, this antibody reacts with all six isoforms of vertabrate actin (Lessard, 1988). Reacts with both globular (G) and fillimentous (F) forms of actin and does not interfere with actin polymerization to form filaments, at a ratio as high as one antibody per two actin monomers. However, this antibody does increase the extent of polymerization when used at a lower ratio of antibody to actin. In addition to labeling myotubes, anti-actin stains myoblasts and fibroblasts. Although clone C4 is prepared as an antibody to chicken gizzard muscles actin, it reacts with actins from all vertebrates, as well as with Dictyostelium discoideum and Physarum polycephalum actins (Lessard, 1988).

Eukaryotic cell lines

Policy information about cell lines

Cell line source(s)	SL1A cell (Spodoptera litura cell line SL1A)
Authentication	Bioresource Collection and Research Center, BCRC No. 960036 - Spodoptera litura cell line SL1A

Mycoplasma contamination	The cell line were not tested for mycoplasma contamination
Commonly misidentified lines (See ICLAC register)	(There is no other name for this cell line)

Animals and other organisms

Policy information about studies involving animals; ARRIVE guidelines recommended for reporting animal research

Laboratory animals	The study did not involve laboratory animals
Wild animals	The study did not involve laboratory wild animals
Field-collected samples	Spodoptera litura were reared in cages and kept in growth chambers at 29 ± 1°C with a photoperiod of 12:12 h (light: dark). Artificial feed was provided with a formula referenced from the previous studies. Snellenius manilae were reared in growth chambers at 29 ± 1°C with a photoperiod of 12:12 h (light: dark). Glucose solution (15%) was used for feeding
Ethics oversight	No ethical approval

Note that full information on the approval of the study protocol must also be provided in the manuscript.
